# Tunable and Passively Mode-Locking Nd_0.01_:Gd_0.89_La_0.1_NbO_4_ Picosecond Laser

**DOI:** 10.3390/molecules26113179

**Published:** 2021-05-26

**Authors:** Shande Liu, Yuqing Zhao, Ke Zhang, Bo Chen, Ning Zhang, Dehua Li, Huiyun Zhang, Yuping Zhang, Lihua Wang, Shoujun Ding, Qingli Zhang

**Affiliations:** 1 College of Electronic and Information Engineering, Shandong University of Science and Technology, Qingdao 266590, China; pepsl_liu@163.com (S.L.); zyq1996yq@163.com (Y.Z.); 17863940297@163.com (K.Z.); bchen_sdust@163.com (B.C.); zhangning09027@163.com (N.Z.); sdust_thz@126.com (H.Z.); sdust_thz@163.com (Y.Z.); 2 School of Science and Engineering of Mathematics and Physics, Anhui University of Technology, Maanshan 243002, China; 3 Key Laboratory of Optoelectronic Materials Chemistry and Physics, Chinese Academy of Sciences, Fuzhou 350002, China; 4 The Key Laboratory of Photonic Devices and Materials, Anhui Institute of Optics and Fine Mechanics, Chinese Academy of Sciences, Hefei 230031, China; zql@aiofm.ac.cn

**Keywords:** Nd:GLNO crystal, tunable laser, mode-locking

## Abstract

A high-quality Nd_0.01_:Gd_0.89_La_0.1_NbO_4_ (Nd:GLNO) crystal is grown by the Czochralski method, demonstrating wide absorption and fluorescence spectra and advantage for producing ultrafast laser pulses. In this paper, the tunable and passively mode-locking Nd:GLNO lasers are characterized for the first time. The tuning coverage is 34.87 nm ranging from 1058.05 to 1092.92 nm with a maximum output power of 4.6 W at 1065.29 nm. A stable continuous-wave (CW) passively mode-locking Nd:GLNO laser is achieved at 1065.26 nm, delivering a pulse width of 9.1 ps and a maximum CW mode-locking output power of 0.27 W.

## 1. Introduction

Ultrafast lasers have been applied in various fields, such as high-precision micro machining, aerospace, and medical diagnostics [1,2]. Benefiting from their low quantum defects, wide gain bandwidth, and simple three-level electronic structure, Yb^3+^-doped laser mediums attract widespread attention in the 1 μm band [3,4,5]. However, the overlap of absorption and emission bands can bring re-absorption loss, resulting in high laser threshold. Compared with Yb^3+^-doped gain mediums, Nd^3+^-doped crystals have no re-absorption loss and are used in low-threshold and high-efficiency ultrafast laser. As is known, the typical gain bandwidth of the Nd^3+^-doped laser materials is narrow, e.g., the gain bandwidth of the Nd:YVO_4_ and Nd:YAG crystals were measured to be only 0.96 and 0.80 nm, respectively [6,7]. For this reason, considerable efforts have been made to explore novel Nd^3+^-doped laser materials with a broad gain bandwidth. The pulse duration of 19.2 ps at 1064 nm was achieved in a passively mode-locked Nd:YVO_4_ laser in 2008 [8]. Mohammad et al. [9]. reported pulse duration of 16 ps generation in a Nd:GdVO_4_ crystal in 2017. He et al. [10]. obtained 3.8 ps pulse duration at a repetition rate of 112 MHz in a Nd:GdYVO_4_ crystal. Previously, theoretical and experimental results have demonstrated that Nd^3+^-doped disordered crystals possess broad emission spectra and are suitable for generating ultrashort lasers [11,12,13].

In the last decade, researchers have invested tremendous enthusiasm into extending Nd^3+^-doped disordered crystals family and exploring their excellent properties. In 2017, a novel disordered crystal Nd_0.01_:Gd_0.89_La_0.1_NbO_4_ (Nd:GLNO) was successfully grown by Anhui Institute of Optics and Fine Mechanics, Chinese Academy of Sciences [14]. Owing to La^3+^ having a relatively large ionic radius in the lanthanide system, the La^3+^-doped disordered crystals exhibit a wider fluorescence bandwidth [15]. Moreover, the difference in ionic radii between La^3+^ and Gd^3+^ ions is small, denoting the Nd:GLNO crystal possesses excellent lattice matching and thermal property [16,17]. The fluorescence lifetime and the radiative lifetime of Nd:GLNO crystal was obtained to be 176.1 μs and 184.5 μs, respectively. The luminescent quantum efficiency of the ^4^F_3/2_ level was estimated to be 95.4% [18]. Ma et al. presented the CW and passively Q-switched Nd:GLNO lasers with Cr^4+^:YAG crystal and PdSe_2_ as saturable absorbers (SAs), respectively, in 2018 and 2020 [15,19]. Unfortunately, the tunable and CW mode-locking Nd:GLNO crystal lasers have not been studied to date.

In this paper, the absorption and florescence spectra of the Nd:GLNO crystal were systematically investigated demonstrating a wide absorption and emission band. A tunable operation Nd:GLNO crystal laser was realized with a tuning range of 34.87 nm from 1058.05 to 1092.92 nm. By employing a semiconductor saturable absorber mirror (SESAM) as SA, a stable CW mode-locking Nd:GLNO crystal laser was achieved, generating the shortest pulse duration of 9.1 ps and the maximum mode-locking output power of 0.27 W.

## 2. Experimental Setup

**Figure 1:**
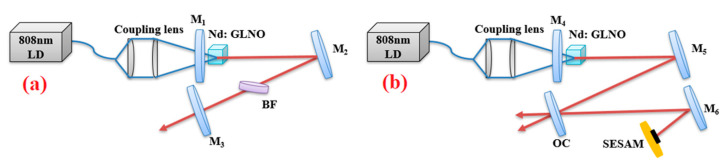
Schematic setups of the Nd:GLNO laser, (**a**) tunable operation; (**b**) CW mode-locking operation.

Figure 1 demonstrates schematic setups of the Nd:GLNO lasers. The 808 nm laser diode was chosen as a pump source with a core diameter of 400 μm and a numerical aperture (NA) of 0.22. The size of the c-cut Nd:GLNO crystal was 2 × 2 × 5 mm^3^. To effectively reduce the influence of thermal effects, the laser crystal was covered with indium and embedded into a copper block. The cooling temperature of the copper block was controlled at 15.5 °C. The total laser cavity length of the mode-locking and tunable lasers was 1.94 m and 0.33 m, respectively. Mirrors M_1_, M_2_, M_4_, M_5_ and M_6_ were all processed with anti-reflection (AR) coating around 808 nm and high-reflection coating (HR, R > 99.9%) at 1030–1100 nm. The curvature radii were R = ∞, R = 200, R = ∞, R = 300 and R = 150 mm, respectively. The output mirror M_3_ was partial transmittances (T) coated at 1030–1100 nm (T = 1, 10, 15%, 25% are available). A quartz birefringent filter (BF) was employed in tunable laser cavity to achieve laser tuning operation. The parameters of the SESAM are as follows: saturable fluence is 90 μJ/cm^2^, absorptance is 1.5%, a modulation depth is 0.8%, damage threshold is 30 mJ/cm^2^, and a relaxation time is 1 ps. A laser power meter (Fieldmax-II, PM10) was used for measuring laser power. The laser output spectra and pulse width of mode-locked Nd:GLNO laser were measured by a spectrometer (Avantes, AcaSpec-3468-NIR256-2.2) and a commercial autocorrelator (APE Pulse Check, 150), respectively. The typical pulse profile and pulse train were recorded by a digital oscilloscope (R&S, RTO 2012) together with a fast InGaAs photon detector (New Focus, 1611). 

## 3. Results and Discussion

**Figure 2:**
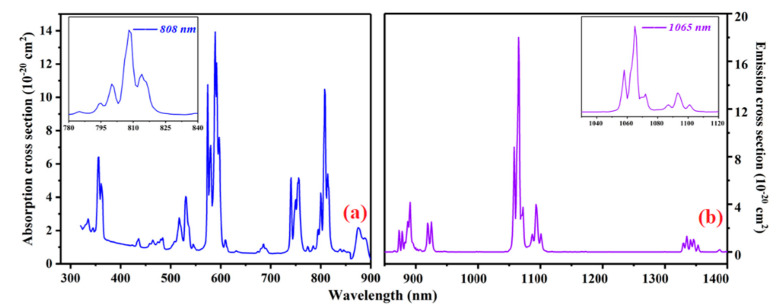
Absorption and fluorescence spectra of the Nd:GLNO crystal. (**a**) Absorption spectra; (**b**) Fluorescence spectra.

Figure 2 presents the absorption and fluorescence spectra of the c-cut Nd:GLNO crystal at room temperature. As shown in Figure 2a, the absorption peak is at 808 nm and FWHM is 13 nm. Based on the equation *σ* = *α*(*λ*)/N_c_, where α is the absorption coefficient (8.97 cm^−1^) and N_c_ is the concentration of Nd^3+^, the maximum absorption cross-section of the Nd:GLNO crystal was calculated to be 10.49 × 10^−20^ cm^2^. Moreover, the stimulated emission cross-section ($\sigma_{\mathrm{em}}$) can be estimated from the fluorescence spectra using the Füchtbauer–Ladenburg equation: $\sigma_{\mathrm{em}}\left(\lambda \right)=\frac{\lambda^{5}I\left(\lambda \right)}{8\pi n^{2}c\tau_{m}\int \lambda I\left(\lambda \right)d\lambda }$ [19,20], where *τ*_m_, *c*, *n*, *I*(*λ*) are the fluorescence lifetime, velocity of light, reflective index and fluorescence intensity, the calculated stimulated emission cross-section of 18 × 10^−20^cm^2^ was relatively large, which was suitable for generating ultrafast laser pulse. 

**Figure 3:**
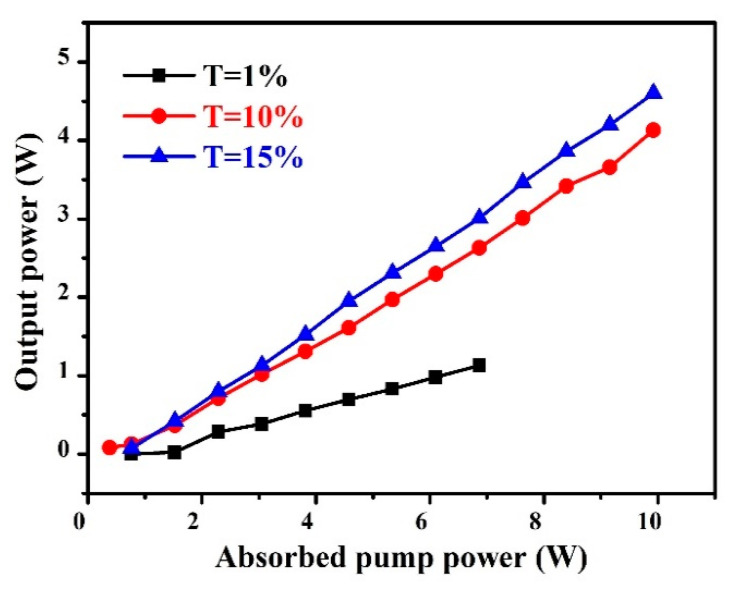
Output power versus absorbed pump power.

**Figure 4:**
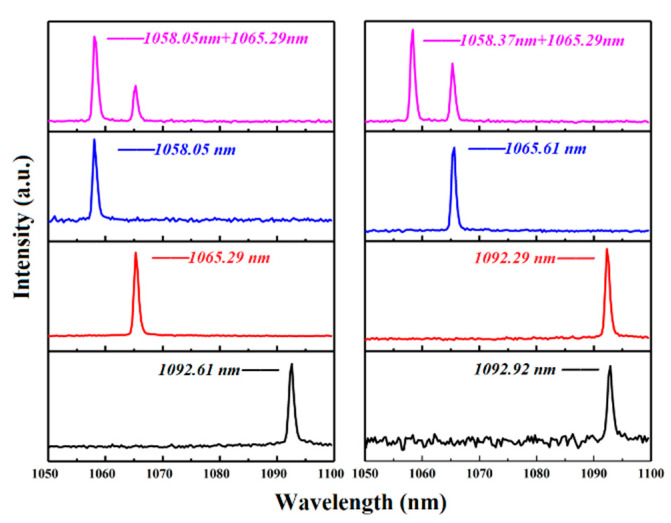
Typical output spectra of the tunable Nd:GLNO laser with T = 1%.

A V-type laser cavity was designed to investigate the CW laser output properties of the Nd:GLNO crystals. Figure 3 displays the relationship between output power and absorbed pump power at different transmittances of output couplers. The maximum CW output power of 4.60 W was achieved with the output mirror of T = 15%, corresponding to an optical-to-optical efficiency of 37.90% and a slope efficiency of 49.67%. Furthermore, the laser output wavelength could be flexibly tuned by carefully varying the angle of the BF. Table 1 records the tuning wavelength and the corresponding output power with the output couplers of T= 1%, 10% and 15%, respectively. As the transmittance increased, the longitudinal mode oscillation in the cavity was suppressed. Therefore, the tuning range was further reduced. The total tuning coverage of the Nd:GLNO crystal laser was 34.87 nm ranging from 1058.05 to 1092.92 nm. Figure 4 demonstrates the typical single wavelength and multi-wavelength spectra of the Nd:GLNO crystal tunable laser.

**Figure 5:**
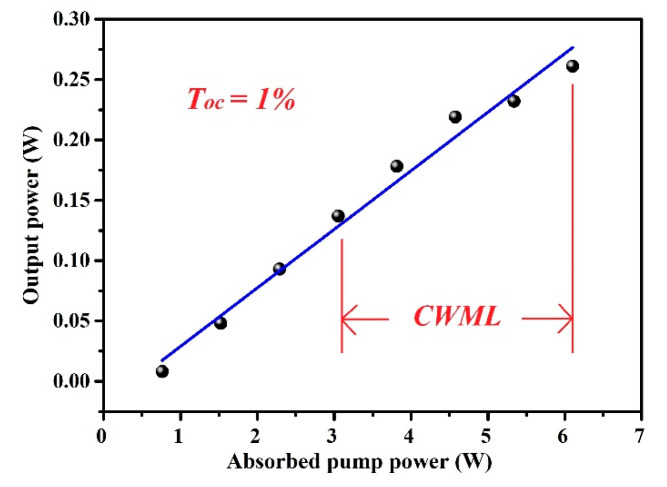
The CW mode-locking output power versus the absorbed pump power.

**Figure 6:**
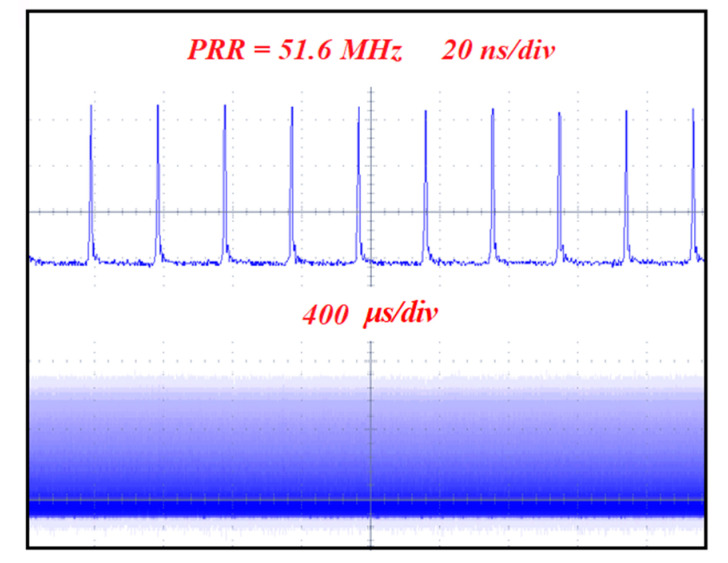
Pulse train of the CW mode-locking Nd:GLNO crystal laser.

**Figure 7:**
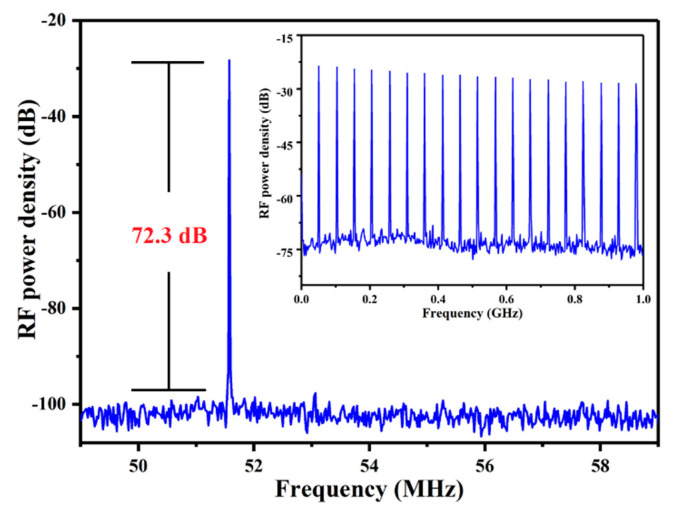
Recorded RF trace.

**Figure 8:**
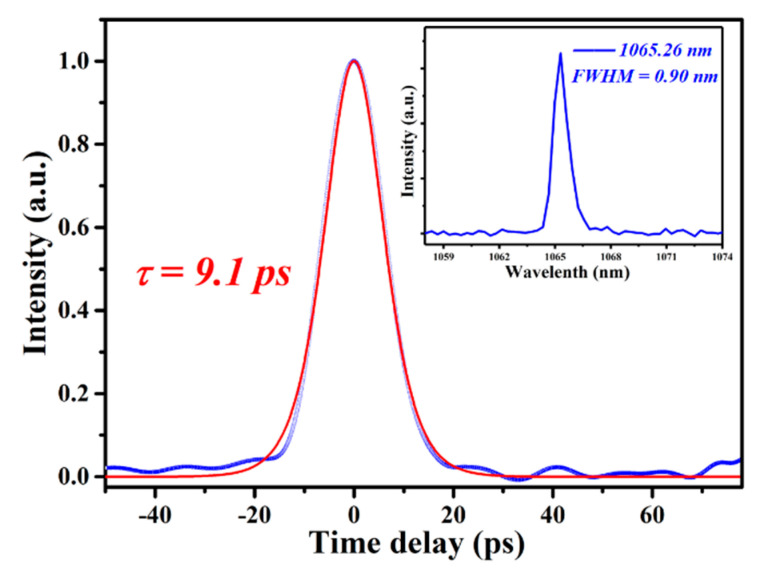
Mode-locking pulse duration and the corresponding spectrum.

To realize the CWML Nd:GLNO laser operation, a Z-type laser cavity was employed as shown in Figure 1b. Ultrafast laser pulse output was achieved using a SESAM. To reduce the intracavity loss and make the SESAM easily saturated, the CWML laser output characteristics were obtained experimentally at the output mirror of T_oc_ = 1%. As shown in Figure 5, the minimum absorbed pump power to suppress Q-switched mode-locking laser was 3.05 W. The maximum CWML laser output power 0.27 W was achieved. The CWML pulse train was measured using a detector and 1 GHz bandwidth oscilloscope. Figure 6 presents the stable mode-locking pulses recorded at nanosecond and microsecond time scales, respectively. The pulse repetition rate (PRR) is 51.6 MHz corresponding to the cavity length of 1.94 m. Figure 7 demonstrates the signal-to-noise ratio of the first beat. The radio frequency spectrum was clean and stable, indicating excellent stability of the mode-locking ultrafast laser. The signal-to-noise ratio was up to 72.3 dB at the fundamental frequency of 51.6 MHz. The FWHM bandwidth of the autocorrelation trace was about 14.0 ps, corresponding to a pulse duration of 9.1 ps by a sech^2^-shape pulse fitting. The mode-locking pulse spectrum was shown in the inset of Figure 8. The central wavelength of the measured pulse was located at 1065.26 nm with a FWHM of 0.9 nm.

## 4. Conclusions

In conclusion, the Nd:GLNO crystal was grown by the Czochralski method and the spectral characteristics at room temperature were discussed. The maximum CW output power of 4.60 W was obtained with the output mirror of T = 15%, corresponding to an optical-to-optical efficiency of 37.90% and a slope efficiency of 49.67%. The tuning coverage of the tunable Nd:GLNO laser was 34.87 nm at T = 1% ranging from 1058.05 to 1092.92 nm. To the best of our knowledge, a picosecond CWML Nd:GLNO laser at 1065.26 nm was experimentally demonstrated using a SESAM as saturable absorber for the first time. The maximum CWML laser output power of 0.27 W was achieved. The Nd:GLNO crystal ultrafast laser produced 9.1 ps mode-locked pulses with pulse repetition rate of 51.6 MHz and a signal-to-noise ratio of 72.3 dB. The results indicated that the Nd:GLNO crystal is a promising Nd^3+^-doped gain medium for generating ultrafast laser pulses.

## Figures and Tables

**Table 1:** Output parameters of the tunable Nd:GLNO crystal laser.

T (%)	Wavelength (nm)	Output Power (W)
1	1058.05	0.83
	1065.29	1.11
	1065.61	0.86
	1091.98	0.58
	1092.29	1.00
	1092.61	0.98
	1092.92	0.79
10	1065.29	4.13
	1092.29	3.03
	1092.61	2.83
15	1065.29	4.60
	1092.29	0.43
	1092.61	0.31

## Data Availability

Data are available upon request to the authors.

## References

[1] Yu T.J., Lee S.K., Sung J.H., Yoon J.W., Jeong T.M., Lee J. (2013). Generation of high-contrast, 30 fs, 1.5 PW laser pulses from chirped-pulse amplification Ti: Sapphire laser. Opt. Express.

[2] Tolstik N., Sorokin E., Sorokina I.T. (2014). Graphene mode-locked Cr:ZnS laser with 41 fs pulse duration. Opt. Express.

[3] Dong L.L., Yao Y.P., Wang Q.G., Liu S.D., Zheng L.H., Xu Y., Li D.H., Zhang H.Y., Zou Z.T., Su L.B. (2018). Tunable and mode-locking Yb,Nd:Sc_2_SiO_5_ femtosecond laser. IEEE Photonics Technol. Lett..

[4] Maddi C., Aswin J.R., Scott A., Aslam Z., Willneff E., Vasala K.N., Adarsh D., Jha A. (2019). Structural, spectroscopic, and excitonic dynamic characterization in atomically thin Yb^3+^-doped MoS_2_, fabricated by femtosecond pulsed laser deposition. Adv. Opt. Mater..

[5] Frédéric D., Sandrine R., Dimitris P., Alain P., Patrice C., Louis D.J., Richard M., Antoine C., Eric M., Patrick G. (2011). On Yb:CaF_2_ and Yb:SrF_2_: Review of spectroscopic and thermal properties and their impact on femtosecond and high power laser performance. Opt. Mater. Express.

[6] Kanchanavaleerat E., Muchy D.C., Kokta M., Sundberg J.S., Sarkies P., Sarkies J. (2004). Crystal growth of high doped Nd:YAG. Opt. Mater..

[7] Zhang H.J., Meng X.L., Zhu L., Wang C.Q., Chow Y.T., Lu M.L. (2000). Growth, spectra and influence of annealing effect on laser properties of Nd:YVO_4_ crystal. Opt. Mater..

[8] Gong M., Yu H., Wushouer X., Yan P. (2008). Passively mode-locked Nd:YVO_4_ picosecond laser with oblique incidence on SESAM. Laser Phys. Lett..

[9] Mohammad N., Tanant W., Arkady M. (2018). Passively mode-locked high power Nd:GdVO_4_ laser with direct in-band pumping at 912 nm. Laser Phys. Lett..

[10] He J.L., Fan Y.X., Du J., Wang Y.G., Liu S., Wang H.T., Zhang L.H., Hang Y. (2004). 4-ps passively mode-locked Nd:Gd_0.5_Y_0.5_VO_4_ laser with a semiconductor saturable-absorber mirror. Opt. Lett..

[11] Feng C., Zhang H.N., Wang Q.P., Fang J.X. (2017). Dual-wavelength synchronously mode-locked laser of a Nd:Y_3_ScAl_4_O_12_ disordered crystal. Laser Phys. Lett..

[12] Liu S.D., Dong L.L., Zhang X., Zheng L.H., Berkowski M., Su L.B., Ren T.Q., Peng Y.D., Hou J., Zhang B.T. (2016). High-power femtosecond pulse generation in a passively mode-locked Nd:SrLaAlO_4_ laser. Appl. Phys. Express.

[13] Qin Z.P., Xie G.Q., Ma J., Ge W.Y., Yuan P., Qian L.J., Su L.B., Jiang D.P., Ma F.K., Zhang Q. (2014). Generation of 103  fs mode-locked pulses by a gain linewidth-variable Nd,Y:CaF_2_ disordered crystal. Opt. Lett..

[14] Ding S.J., Zhang Q.L., Luo J.Q., Liu W.P., Lu W.C., Xu J.R., Sun G.H., Sun D.L. (2017). Crystal growth, structure, defects, mechanical and spectral properties of Nd_0.01_: Gd_0.89_La_0.1_NbO_4_ mixed crystal. Appl. Phys. A.

[15] Ma Y.F., Sun H.Y., Peng F., Ding S.J., Yu X., Zhang Q.L. (2018). Diode-pumped continuous-wave and passively Q-switched Nd:GdLaNbO_4_ laser. Opt. Mater. Express.

[16] Takei H., Tsunekawa S. (1977). Growth and properties of LaNbO_4_ and NdNbO_4_ single crystals. J. Cryst. Growth.

[17] Hakimova L., Kasyanova A., Farlenkov A., Lyagaeva J., Medvedev D., Demin A., Tsiakaras P. (2019). Effect of isovalent substitution of La^3+^ in Ca-doped LaNbO_4_ on the thermal and electrical properties. Ceram. Int..

[18] Ding S.J., Zhang Q.L., Sun D.L., Peng F., Liu W.P., Luo J.Q., Sun G.H. (2018). Spectroscopic properties of Nd:Gd_0.89_La_0.1_NbO_4_ mixed laser crystal. J. Lumin..

[19] Ma Y.F., Zhang S.C., Ding S.J., Liu X.X., Yu X., Peng F., Zhang Q.L. (2020). Passively Q-switched Nd:GdLaNbO_4_ laser based on 2D PdSe_2_ nanosheet. Opt. Laser Technol..

[20] Sokólska I., Heumann E., Kück S., Lukasiewicz T. (2000). Laser oscillation of Er^3+^: YVO_4_ and Er^3+^, Yb^3+^: YVO_4_ crystals in the spectral range around 1.6 μm. Appl. Phys. B.

